# The Effect of a Health Education Program on Knowledge, Medication Adherence, and Glycosylated Hemoglobin in Rural Patients with Type 2 Diabetes

**DOI:** 10.2174/0115733998332358240923112144

**Published:** 2024-11-29

**Authors:** Zeinab Elyasi, Nasrin Ghiasi, Ali Khorshidi, Salar Bakhtiyari, Mohsen Jalilian

**Affiliations:** 1 Department of Public Health, Faculty of Health, Ilam University of Medical Sciences, Ilam, Iran;; 2 Department of Epidemiology, Faculty of Health, Ilam University of Medical Sciences, Ilam, Iran;; 3 Department of Clinical biochemistry, School of Medicine, Ilam University of Medical Sciences, Ilam, Iran

**Keywords:** Health education, type 2 diabetes, self-regulation theory, diabetes management, medication adherence, HbA1c, rural health

## Abstract

**Introduction:**

Improving medication adherence plays a crucial role in preventing adverse complications of type 2 diabetes.

**Objectives:**

This study aimed to determine the effect of a health education program on knowledge, medication adherence, and HbA1c in rural patients with type 2 diabetes.

**Methods:**

This experimental study was conducted in Ilam County, Iran. Participants were selected *via* multistage cluster sampling and randomly assigned to either the intervention group (n=43) or the control group (n=42). The intervention group underwent a one-month educational program based on self-regulation theory, consisting of 12 sessions, while the control group received no educational intervention. Data were collected at baseline, three months, and six months using a diabetes knowledge test (DKT), the Iranian version of the Morisky Medication Adherence Scale-8 (IVMMAS-8), and laboratory measurements of HbA1c. Statistical analysis was performed using SPSS version 16, employing chi-square tests, independent sample t-tests, repeated-measures ANOVA, and Bonferroni correction at a significance level of 0.05.

**Results and Discussion:**

After 3 and 6 months, the intervention group showed significant improvements in diabetes knowledge and medication adherence and a significant reduction in HbA1c levels compared to the control group (*p*<0.001). No significant changes were observed in the control group over time (*p*>0.05).

**Conclusion:**

This study demonstrated that health education based on self-regulation theory can effectively increase knowledge, improve treatment adherence, and decrease HbA1c levels in rural patients with type 2 diabetes.

## 1 INTRODUCTION

Type 2 diabetes imposes a substantial burden of illness and death on public health, clearly ranking among the most formidable global health challenges in the 21^st^ century [1-3]. According to a 2021 report by the International Diabetes Federation (IDF), type 2 diabetes has the highest prevalence (16.2%) in the Middle East and North Africa region (MENA). The MENA region also had the highest percentage (24.5%) of diabetes-related deaths among people of working age. Predictions indicate that the prevalence of type 2 diabetes in these regions will increase to 17.6% by 2030 and 19.3% by 2045 [4]. In Iran, there are approximately 5.5 million diabetic patients, with the prevalence among adults expected to rise from 9.1% in 2021 to 12.3% in 2035 [4].

Despite significant advancements and concerted efforts toward evidence-based diabetes management, poor glycemic control remains widespread [3, 5]. Enhancing metabolic control through dietary interventions, physical activity, and adherence to medications can markedly reduce the risk of complications [6, 7].

Managing type 2 diabetes necessitates lifestyle adjustments and continuous learning to effectively engage in long-term self-care practices [8-10]. Patient education, combined with adherence to prescribed medications and dietary regimens, empowers individuals to assume greater responsibility for their own care [11]. Non-adherence and non-compliance with prescribed treatments can result in heightened complications and adverse effects related to the disease [12]. However, evidence suggests weak adherence to medication regimens among diabetic patients [6, 10]. Measurement of glycosylated hemoglobin (HbA1c) is a widely used method to assess both control and adherence to treatment in type 2 diabetes. HbA1c levels are considered the gold standard for evaluating long-term diabetes management [13, 14].

In recent years, numerous studies have emphasized encouraging patients to diligently follow treatment regimens by enhancing their knowledge of self-care behaviors and adopting lifestyle changes [15-17]. Therefore, identifying solutions to aid in type 2 diabetes control is of paramount importance, with education and empowerment being fundamental for enhancing the disease control capabilities of diabetic individuals [18, 19]. Empowerment occurs when individuals acquire essential information about their condition to make informed decisions and effectively exercise self-control. It also involves having supportive environments for decision-making and possessing the experience to evaluate decision outcomes [15, 20, 21].

Frameworks, such as Albert Bandura's self-regulation theory, can enhance the effectiveness of these interventions in empowering patients to cope with their illness [22-24]. Bandura's theory operates through three major subfunctions of self-regulation: self-monitoring, self-judgment/re-evaluation, and self-reaction [25, 26]. Recent studies have highlighted the efficacy of self-regulation theory in adult education. This theory empowers learners to set personal goals, monitor their progress, and adjust their strategies to optimize learning outcomes. Self-regulation promotes active engagement and accountability, which is crucial for adult learners who often juggle multiple responsibilities [27, 28]. As mentioned above, this study aimed to investigate the effect of a health education program on knowledge, medication adherence, and HbA_1_c in rural patients with type 2 diabetes.

## 2 METHODS

### 2.1 Study Design and Participants

This was an experimental study conducted on 85 rural patients with type 2 diabetes in Ilam County and western Iran from April to November, 2023. The study population consisted of all patients with type 2 diabetes attending rural comprehensive health service centers affiliated with Ilam County.

### 2.2 Sampling

A multistage cluster sampling method was utilized for sampling. Ilam County has six rural comprehensive health service centers located in Haft Cheshmeh, Chalsara, Mehrabad, Mishkhas, Shahid Keshvari Village, and Pakal villages. Shahid Keshvari Village Comprehensive Health Service Center was selected as the intervention group, and Mishkhas Comprehensive Health Service Center was chosen as the control group. Forty-five individuals were selected from each study arm based on the inclusion criteria. The sample size was calculated using the following formula:


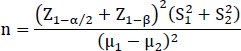


### 2.3 Inclusion and Exclusion Criteria

Considering that the history of the disease influences the onset of its complications, the study sample was composed of patients who did not exhibit serious disease-related complications. Therefore, the study sample was selected carefully based on the following inclusion and exclusion criteria:

The inclusion criteria included age between 30 and 59 years, definitive diagnosis of type 2 diabetes, HbA1c levels above 7%, use of oral antidiabetic drugs, ability to participate in educational programs, basic literacy (minimum fifth grade), possession of a smartphone for follow-up, and completion of informed consent forms. The exclusion criteria included insulin injection, physical complications of the disease, such as foot ulcers, severe kidney and eye problems, gestational diabetes, and participation in a concurrent similar study (Fig. **1**).

### 2.4 Intervention

A one-month educational program was conducted for the intervention group at the education hall of the Shahid Keshvari Village Comprehensive Health Service Center and village mosques. Before starting the intervention program, a session was conducted with the patients to discuss the program schedule and its location. During this session, it was agreed that the classes would generally last for 60 minutes and be held in the afternoons to accommodate patient attendance. A total of 12 sessions, each lasting 60 minutes, were conducted in group settings, along with individual counseling sessions.

Group sessions were structured to empower patients to use self-regulation processes to improve adherence to medication and manage type 2 diabetes. Individual counseling sessions aimed to assist patients in setting realistic goals, overcoming barriers to adherence, and improving disease management. The intervention was implemented by an experienced health educator to ensure consistency and uniformity throughout the sessions. Health communication principles were employed during program implementation to foster interactive and bidirectional communication between participants and health educators.

In addition to face-to-face education, a messaging group titled “Living with Diabetes” was created on the “Bale” messaging application (an Iranian messaging application), and all intervention group participants were invited to join. Educational podcasts were uploaded to the group three times a week. Moreover, to maintain the intervention effect, educational messages regarding blood glucose control and medication adherence were sent to the intervention group every 15 days during the 3-month and 6-month follow-up periods.

It is worth noting that during the implementation of this educational program, the control group received only routine education provided at comprehensive health service centers. Furthermore, after collecting the data from the final phase, a pamphlet and a booklet on diabetes knowledge were delivered to the control group.

### 2.5 Instruments

Data collection for this study involved the use of questionnaires completed through participant interviews. To assess knowledge, the Diabetes Knowledge Test (DKT) developed by Fitzgerald was utilized [29]. This test evaluates general knowledge about diabetes and its treatment. In this study, only the general section consisting of 14 questions suitable for adults with type 1 and type 2 diabetes was used. According to the DKT scoring protocol, items were scored as 1 for correct answers and 0 for incorrect answers. The resulting score was then reported as a percentage out of 100. Accordingly, scores of 76-100 were classified as high knowledge, scores of 50-75 as moderate knowledge, and scores below 50 as low knowledge.

Additionally, to measure medication adherence, the Iranian version of Morisky Medication Adherence Scale-8 (IVMMAS-8) was used [30]. This scale consists of 8 items, with the first 7 items answered as yes or no. A score of 0 was assigned for each yes answer, and a score of 1 was assigned for each no answer. Item 8 was designed on a 5-point Likert scale (never, rarely, sometimes, often, and always), and scores of 1 were given for never, rarely, and sometimes, 0.75 for often, and 0 for always. A score of less than 6 points indicated poor adherence, 6 or 7 points indicated medium adherence, and 8 points indicated complete adherence [30].

Furthermore, for measuring HbA1c levels, patients were referred to the laboratory, and after receiving the test results, they were recorded on a checklist.

### 2.6 Data Analysis

Data for the study were collected in three stages: pretest, 3 months later, and 6 months later, through participant interviews. SPSS version 16 was used for the data analysis. Chi-square tests, independent sample t-tests, repeated-measures ANOVA, and Bonferroni post hoc correction were used for data analysis at a significance level of 0.05.

### 2.7 Ethical Aspects

This study was approved by the Research Ethics Committee of Ilam University of Medical Sciences with the code IR.MEDILAM.REC.1401.131.

## 3 RESULTS

Eighty-five patients with type 2 diabetes participated in this study, with a mean age of 49.3 ± 7.46 years. The dropout rate for the study was 5.5%. Further details are provided in Table **1**. The results of the chi-square test showed that the groups were homogeneous in terms of the distribution of demographic variables at the pretest (*p* > 0.05).

Additionally, the independent sample t-test results indicated no significant difference between the groups in terms of the mean scores of knowledge, medication adherence, or HbA_1_c at the pretest (*p* > 0.05).

As mentioned in Table **2**, the results of the Greenhouse- Geisser test indicated that the educational intervention significantly affected the mean scores of knowledge [F[1.22, 126.53] = 26.22, *p* < 0.00^1^], medication adherence [F[1.26, 151.83] = 28.26, *p* < 0.00^1^], and HbA1c [F[1.10, 136.64] = 10.98, *p* < 0.00^1^] in the intervention group compared to the control group.

Furthermore, considering the interaction between the variables of knowledge [F[1.21, 126.53] = 37.21, *p* < 0.001], medication adherence [F[1.26, 151.83] = 76.48, *p* < 0.001], and HbA1c [F[1.15, 136.64] = 15.01, *p* < 0.001], as well as the group [intervention and control], separate analyses were conducted at the group variable level. Changes in mean knowledge, medication adherence, and HbA1c in the intervention and control groups over time are presented in Tables **3** and **4**.

The trend of change in mean knowledge score and medication adherence at 3 and 6 months compared to baseline in the intervention group was significant (*p* < 0.001), but the change at 6 months compared to 3 months was not significant (*p* > 0.05). Conversely, the trend of changes in the mean of these variables in the control group over time was not significant (*p* > 0.05).

Additionally, the impact of variables, such as age, sex, marital status, occupation, and disease history, on the relationship between education and knowledge in both the intervention and control groups was not significant (*p* < 0.05).

## 4 DISCUSSION

This study revealed significant changes in type 2 diabetes knowledge, medication adherence, and HbA1c in the intervention group, indicating the importance of self-regulation processes in diabetes management. There was a notable increase in diabetes knowledge scores in the intervention group, affirming the effectiveness of education in enhancing awareness and knowledge acquisition. These findings align with similar studies; for instance, Moradi *et al.* (2018) demonstrated the effectiveness of educational intervention using short message service (SMS) in increasing diabetes knowledge [31]. Similarly, Nazir *et al.* (2020) reported the positive impact of a medication management program on increasing knowledge related to diabetes, medication adherence, and quality of life among study participants [32]. It appears that enhancing knowledge, along with learning self- regulation strategies, can play a significant role in improving medication adherence, quality of life, and clinical outcomes in patients. Enhancing knowledge and employing self-regulation strategies appear crucial in improving medication adherence, quality of life, and clinical outcomes in patients. Thus, empowering patients with knowledge can enhance their engagement and interaction in their treatment process, leading to better outcomes compared to less engaged patients.

The results of the present study indicated an increase in medication adherence in type 2 diabetic patients in the intervention group after implementing the educational program. The average adherence score in the intervention group increased significantly at 3 and 6 months after the completion of the educational program. Self-regulation processes were used to activate patients to accept responsibility for controlling and managing their disease. Additionally, the use of individual counseling approaches and sending reminder messages significantly increased patients' adherence to the medication regimen. This finding is consistent with the results of a study by Schoenthaler *et al.* (2020) on the effectiveness of mHealth-based educational interventions for improving medication adherence in African American patients with uncontrolled hypertension and type 2 diabetes [33]. Moreover, the results of the present study regarding medication adherence are in line with those of Parsaei *et al.* (2019), who investigated the impact of education on medication adherence in elderly patients with type 2 diabetes [34]. Furthermore, Raj *et al.* (2020) reported similar results in their study on the effect of behavioral intervention on medication adherence in elderly Indian patients with noncommunicable diseases at three and six months after the intervention [35]. Overall, it appears that educational interventions are well-received for enhancing medication adherence among patients with type 2 diabetes. Such interventions can serve as a suitable, straightforward, and effective method to help patients comprehend the significance of adhering to their medications.

Based on the findings of this research, education based on self-regulation theory was effective in reducing HbA1c levels in diabetic patients. This finding is similar to the results observed in studies by Wang *et al.* (2014), Seuring *et al.* (2019), and Hajloo *et al.* (2021), who reported a reduction in HbA1c after educational intervention [36-38].

## CONCLUSION

According to the results of the present study, mean diabetes knowledge and medication adherence increased in the intervention group at 3 and 6 months post-intervention, with HbA1c levels decreasing to desirable control ranges. These findings highlight the effectiveness of education based on self-regulation theory in improving clinical outcomes by enhancing diabetes knowledge and medication adherence. Given that medication adherence hinges on patient-driven behaviors, these behaviors can significantly influence patients' health status, potentially reducing complications and treatment costs.

Although changes in mean knowledge, medication adherence, and HbA1c at 6 months compared to 3 months were not statistically significant in this study, the averages of these variables remained within acceptable ranges. This underscores the importance of carefully planning reminder strategies to sustain the intervention's effects, particularly in the months following the educational program.

Based on the findings of the current study, future research should focus on refining and tailoring reminder systems to enhance the sustainability of intervention effects. Exploring various frequencies and methods of reminders could prolong the observed benefits post-intervention. Additionally, extending follow-up periods beyond six months would provide valuable insights into the long-term impact on diabetes knowledge, medication adherence, and HbA1c levels. Evaluating the cost-effectiveness of self-regulation theory-based educational interventions compared to standard care would offer essential data for informing healthcare policy decisions.

## STRENGTHS AND LIMITATIONS

This study utilized educational intervention as a high- cost, effective approach. Activating self-regulation processes to increase patients' responsibility in managing their disease was another of its strengths. Among the limitations of this study not examining nutritional behaviors as a contributing factor to HbA1c levels should be noted. Another limitation was the omission of family support in patients' adherence to treatment.

## Figures and Tables

**Fig. (1):**
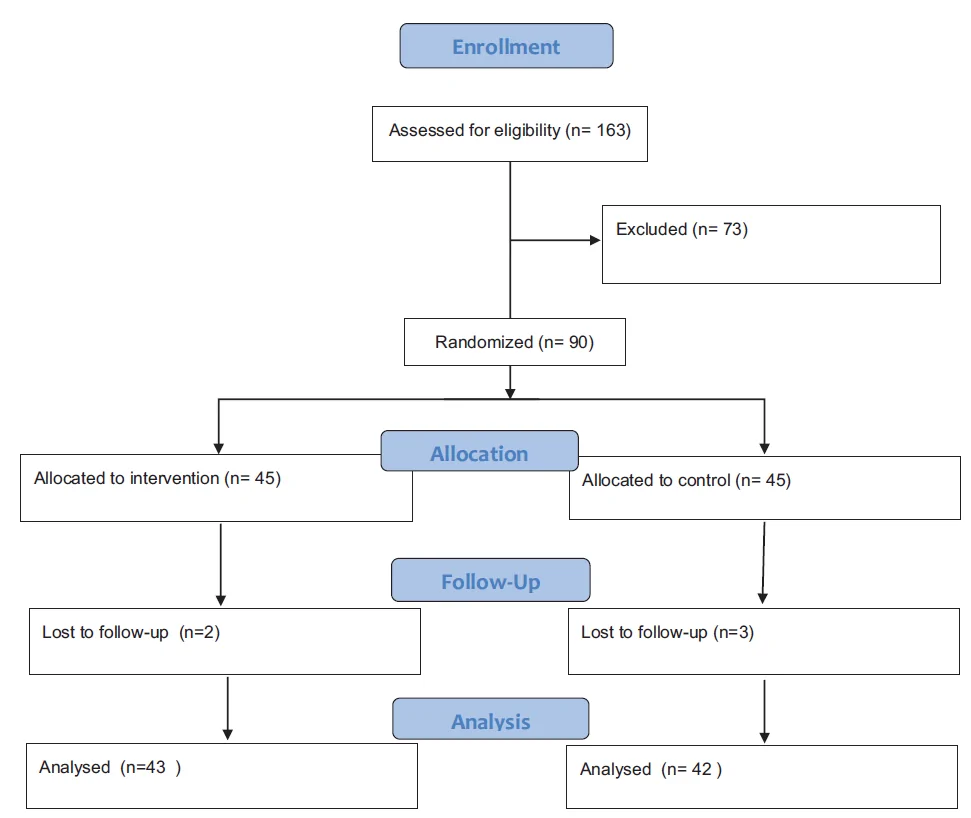
Study flow diagram.

**Table 1:** Participant demographic characteristics.

-		**Intervention [N%]**	**Control [N%]**	***p*-value**
**Gender**	Women	30 [69.7]	28 [66.6]	0.92
	Men	13 [30.2]	14 [33.4]	
**Education level**	Elementary	16 [37.2]	23 [54.7]	0.29
	Guidance	15 [34.8]	11 [26.2]	
	High sSchool	10 [32.2]	6 [14.2]	
	University	2 [4.6]	2 [4.8]	
**Job**	Employed	-	2 [4.8]	0.355
	Unemployed	13 [30.2]	14 [33.4]	
	Housekeeper	30 [69.7]	26 [61.9]	
**Marital status**	Married	41 [95.4]	39 [92.8]	0.645
	Single	2 [4.6]	3 [7.2]	
**Family size**	≤ 5	27 [62.7]	24 [57.1]	0.666
	> 5	16 [37.2]	18 [42.9]	

**Table 2:** Comparison of knowledge, medication adherence, and HbA1c between groups by time.

-	**Intervention**			**Control**			***p*-value**
	**Pretest**	**3 months**	**6 months**	**Pretest**	**3 months**	**6 months**	
**Knowledge**	7.63±1.6	9.37±1.3	9.63±1.2	6.97±2.4	6.81±2.5	7.11±2.1	<0.01
**Medication adherence**	4.06±1.4	5.64±1.24	5.94±1.12	3.92±1.04	3.76±.95	3.59±0.93	<0.01
**HbA_1_c**	9.47±1.77	8.08±2.17	7.79±2.07	8.91±1.51	8.99±1.61	9.06±2.06	<0.01

**Table 3:** Changes in the mean score of knowledge, medication adherence, and HbA1c in the intervention group by time.

-		**Mean Difference**	**Standard Error**	**Confidence Interval (CI 95%)**	***p*-value**
**Knowledge**	3 months	1.74	20.194	1.26 to 2.23	<0.01
	6 months	2	0.223	1.443 to 2.557	<0.01
	3 months to 6 months	0.256	0.145	-0.618 to 0.107	>0.05
**Medication adherence**	3 months	1.576	0.233	0.994 to 2.157	<0.01
	6 months	1.878	0.198	2.372	<0.01
	3 months to 6 months	0.302	0.128	-0.017 to 0.622	>0.05
**HbA_1_c**	3 months	-1.395	0.326	-2.208 to -0.582	<0.01
	6 months	-1.684	0.343	-2.540 to -0.827	<0.01
	3 months to 6 months	-0.288	0.181	-0.162 to 0.740	>0.05

**Table 4:** Changes in the mean score of knowledge, medication adherence, and HbA1c in the control group by time.

-		**Mean Difference**	**Standard Error**	**Confidence Interval (CI 95%)**	***p*-value**
**Knowledge**	3 months	-0.167	0.322	-0.662 to 0.995	> 0.05
	6 months	0.143	0.296	-0.597 to 0.882	> 0.05
	3 months to 6 months	-0.310	0.169	-0.730 to 0.111	> 0.05
**Medication adherence**	3 months	-0.167	0.086	-0381 to 0.048	> 0.05
	6 months	-0.333	0.162	-0.737 to 0.070	> 0.05
	3 months to 6 months	-0.167	0.149	-0.206 to 0.539	> 0.05
**HbA_1_c**	3 months	0.076	0.083	-0.284 to 0.132	> 0.05
	6 months	-0.150	0.248	-0.769 to 0.469	> 0.05
	3 months to 6 months	-0.074	0.219	-0.620 to 0.743	> 0.05

## Data Availability

The data file, materials, and consent form are available and can be provided upon request.

## LIST OF ABBREVIATIONS

IDF: International Diabetes Federation
MENA: Middle East and North Africa
HbA1c: Glycosylated Hemoglobin
DKT: Diabetes Knowledge Test
IVMMAS-8: Iranian version of Morisky Medication Adherence Scale-8

## STANDARDS OF REPORTING

CONSORT guidelines were followed.
